# An Account of a Successful Operation for Erectile Tumour after the Manner of Lallemand

**Published:** 1838-03-14

**Authors:** J. Pancoast

**Affiliations:** Lecturer on Anatomy, &c., Surgeon to the Philadelphia Hospital, &c.


*An Account of a Successful Operation for Erectile Tumour after the manner of Lallemand.* By J. PANCOAST, M. D., *Lecturer on Anatomy, &c., Surgeon to the Philadelphia Hospital, &c.*

DRS. BIDDLE *and* CLYMER.

Gentlemen: In the Archives Generales, for 1835, Professor Lallemand, of Montpelier, has reported several cases of Erectile tumour, successfully treated by a new method.

One was a case of Erectile tumour, involving the gums and alveoli of the lower jaw, in a child, which he removed much in the same manner as Bell's method for the cure of Epulis, with the knife, mallet, chisel, and trephine; arresting the hemorrhage with the actual cautery, the wounds of the soft parts being brought together with the twisted suture and bandages. In a second case of elongated erectile tumour of the upper lip and edges of the nostrils, he excised a triangular portion from the lip, and brought the parts together with the twisted suture.

In a third case of erectile tumour of the cheek, two inches long, and one and a half broad, he effected a cure by an incision, through the long axis of the tumour, bringing the edges together, with four needles, surrounded by numerous coils of thread, as in the common operation for hare lip. Much inflammation, pain, and swelling followed in all these, spreading from the tracts of the needles, into the whole mass of the tumour, effacing its cells, and converting it into a sort of fibro-cartilage, with a white, smooth, shining surface, like the cicatrix resulting from a burn. A fourth case of erectile tumour of the shoulder, three inches long, and two broad, he treated by acupuncturation, without incision, trusting to the inflammation developed along the tracts of the needles to effect a cure. He passed 12 needles at a time, through one portion of the tumour, covering the space between the needles with many turns of a waxed thread. Three days after, he treated another portion of the tumour in the same manner, leaving the needles in for about eight days, till an "agglutinative inflammation" had been fairly excited.

The treatment of this case continued for nearly three months, during which period one hundred and twenty needles had been successively passed through different portions of the tumour, without the loss of a single tea spoonful of blood, and without the general health of the patient suffering for more than three or four days in all.

The result of this case convinced M. Lallemand that the incision and excision in the two preceding cases were unnecessary, and determined him subsequently to trust to acupuncturation alone, to effect a cure.

In May, 1837, I was called to see a female infant of Mr. K. in Union street, seven months old. It had an erectile tumour on the back, just below the right scapula, two and three quarter inches long, and two broad, about three fourths of an inch thick, involving the skin and subcutaneous cellular tissue, and forming a very red prominent tumour on the back. The tumour had made its appearance, as a small red spot, shortly after the birth of the child and was enlarging rapidly. The infant was weak and delicate, so much so as to render it very probable that it would sink under the common operation of extirpating the tumour, either with the ligature or the knife.

May 23, 1837. Assisted by Dr. J. Randolph, I introduced twelve acupuncture needles through the tumour; eight of these were passed through the base of the tumour, in its short diameter; and four at right angles, over the others in the direction of its long axis. I threw several turns of a waxed thread loosely over each needle, and several turns more loosely still, under the points and base of the needles; I bent a strap of adhesive plaster nicked at its outer border, around the tumour, to keep the needles from irritating the sound skin. I laid thick compresses on each side of the tumour to protect it from pressure, and surrounded the whole with a bandage. The patient appeared to suffer scarcely any inconvenience from the needles till the eighth day, when she became fretful; on the ninth, the bandage was undone and the needles were withdrawn. Scarcely a drop of blood had as yet been lost. Suppuration had taken place along the tracts of the needles—and the threads covering each needle, and those surrounding the base of the tumour had caused a little superficial ulceration. Simple dressings were first applied, changed a few days after for mild Basilicon ointment, to promote the suppurating discharge. No other needles were introduced, and in three months from the time of the operation, the tumour had lost all its redness, with the exception of a few points, had greatly diminished in size and thickness, was semi-fibro-cartilaginous to the touch, and had a polished shining surface like the cicatrix of a burn. I proposed to remove the remaining red points with lunar caustic, but the parents of the child preferred waiting to see if they were disposed to enlarge. They remain at the present moment, without having undergone any enlargement. During the cure of the tumour the health of the child was not in the least impaired by the treatment. This case, and one reported by my friend Dr. Coates, as under treatment, in the Examiner, are the only instances that have come to my knowledge, in which Lallemand's ingenious operation has been practised in this country.

The idea of curing erectile tumours, by effacing the cells by "agglutinative inflammation," is not original with Lallemand, but the method only is his of producing it by acupuncturation. I have repeatedly, and so have several others in the city, cured small *superficial* erectile tumours, by vaccination over the surface.

The mere injection of the cellular structure of an erectile tumour, with an irritating fluid, (by insinuating a tube through the skin, which I found easy to accomplish in the dead body,) would necessarily produce this agglutinative inflammation, and I think effect a cure with less inconvenience even than the use of the needles. I intend to give this plan a trial on the first occasion. Yours, &c.

J. Pancoast.

Philadelphia, March 3, 1838.

